# Xanthine oxidase inhibition by febuxostat attenuates stress-induced hyperuricemia, glucose dysmetabolism, and prothrombotic state in mice

**DOI:** 10.1038/s41598-017-01366-3

**Published:** 2017-04-28

**Authors:** Maimaiti Yisireyili, Motoharu Hayashi, Hongxian Wu, Yasuhiro Uchida, Koji Yamamoto, Ryosuke Kikuchi, Mohammad Shoaib Hamrah, Takayuki Nakayama, Xian Wu Cheng, Tadashi Matsushita, Shigeo Nakamura, Toshimitsu Niwa, Toyoaki Murohara, Kyosuke Takeshita

**Affiliations:** 1 Department of Cardiology, Nagoya University Graduate School of Medicine, Nagoya, Japan; 2 Department of Clinical Laboratory, Nagoya University Hospital, Nagoya, Japan; 3 Department of Blood Transfusion, Nagoya University Hospital, Nagoya, Japan; 4 Department of Pathology, Nagoya University Hospital, Nagoya, Japan; 5 Department of Blood Transfusion, Aichi Medical University Hospital, Nagakute, Japan; 6 Department of Cardiology, Shanghai General Hospital, Shanghai Jiao Tong University School of Medicine, Shanghai, China; 7 Faculty of Health and Nutrition, Shubun University, Ichinomiya, Aichi Japan

## Abstract

Chronic stress is closely linked to the metabolic syndrome, diabetes, hyperuricemia and thromboembolism, but the mechanisms remain elusive. We reported recently that stress targets visceral adipose tissue (VAT), inducing lipolysis, low-grade inflammation with production of inflammatory adipokines, metabolic derangements such as insulin resistance, and prothrombotic state. In the present study, we hypothesized the involvement of VAT xanthine oxidoreductase (XOR), a source of reactive oxygen species (ROS) and uric acid (UA) in the above processes. Restraint stress in mice resulted in upregulation of XOR and xanthine oxidase activity, accumulation of ROS in VAT as well as liver and intestine, increase in serum UA levels, upregulation of NADPH oxidase subunits and downregulation of antioxidant enzymes. Immunohistochemistry and RT-PCR analysis also showed that restraint stress induced VAT monocyte accumulation and proinflammatory adipokine production, resulting in reduced insulin sensitivity and induction of plasminogen activator inhibitor-1 and tissue factor in VAT. Treatment with febuxostat, a potent XO inhibitor, suppressed stress-induced ROS production and VAT inflammation, resulting in improvement of serum UA levels, insulin sensitivity, and prothrombotic tendency. Our results suggest that stress perturbs glucose and UA metabolism, and promotes prothrombotic status, and that XO inhibition by febuxostat might be a potential therapy for stress-related disorders.

## Introduction

The relationship between hyperuricemia and stress has been discussed for a long time^1^. A study in occupational health also suggested the involvement of stressful conditions, such as shift-work, is in the incidence of hyperuricemia^2^. Chronic psychological stress in modern lifestyle is closely linked to incidence of metabolic syndrome (MetS), diabetes mellitus, and thromboembolism^3^. It has been hypothesized that Mets and uric acid dysmetabolism share a common mechanism under stressful condition.

Recent studies from our laboratories indicated that visceral adipose tissue (VAT) is one of the targets of psychological stress-induced disorders, similar to Mets, and demonstrated that two-week intermittent restraint stress in a murine model evoked chronic inflammation of the adipose tissue followed by lipolysis in VAT with free fatty acid (FFA) release and TLR-4 stimulation^4^. Furthermore, the stress-induced low-grade inflammation of the VAT produced inflammatory adipokines, including tumor necrosis factor-α (TNF-α), interleukin-6 (IL-6), and monocyte chemoattractant protein-1 (MCP-1), and exacerbated monocyte accumulation, and subsequently resulted in impaired insulin sensitivity and prothrombotic state, with increase in the levels of tissue factor (TF) and plasminogen activator inhibitor-1 (PAI-1)^4, 5^, similar to the events described in the pathophysiological process of MetS^6^. There is a growing evidence to suggest that chronic psychological stress promotes the production of reactive oxygen species (ROS) throughout the body^7^. We also identified VAT as a major source of ROS in connection with this inflammation and therapeutic target under the stressful condition^8, 9^.

Xanthine oxidoreductase (XOR) is a molybdopterin-containing enzyme that catalyzes the oxidation of hypoxanthine to xanthine and finally to uric acid, and exists in two forms: xanthine dehydrogenase (XDH), which prefers NAD^+^ as electrons acceptor, and xanthine oxidase (XO), which is derived from XDH by posttranslational modification, and generates electrons that are transferred directly to molecular oxygen, leading to the formation of the ROS superoxide^10^. XOR expression level and enzymatic activity are high in VAT, similar to liver and intestine in the mouse^11, 12^. XOR expression is induced by the inflammatory cytokines such as interleukin-1, IL-6, TNF-α^13^. XOR expression in adipose tissue is enhanced and produce uric acid in an obese murine model^12^. Increased ROS accumulation in VAT, which is accompanied by increase in nicotinamide adenine dinucleotide phosphate (NADPH) oxidase (NOX) subunits and decrease in antioxidant enzymes, has been recognized as the early instigator and potential therapeutic target of Mets^14^. Since NOX and XO activate each other through the production of superoxide anion^10, 15^, XO would also play a critical role in free radical production in VAT under stressful condition as well as Mets.

Previous studies suggested the involvement of adipose XOR in stress-induced ROS production and dysmetabolism of uric acid, and demonstrated that febuxostat, a highly potent inhibitor of XOR^16^, inhibited the conversion of xanthine to uric acid and suppressed the toxic overproduction of ROS. The aim of the present study was to determine whether febuxostat can suppress stress-induced inflammation and ROS production in VAT, liver and intestine, improve insulin sensitivity, and minimize prothrombotic tendency. To study the mechanisms of such actions, we measured the expression of XOR, ROS production, and enzymatic activity in VAT, liver and intestine, and serum uric acid levels in a murine restraint stress model.

## Results

### Febuxostat reduced plasma uric acid level and adipose tissue xanthine oxidoreductase activity in stressed mice

**Figure 1:**
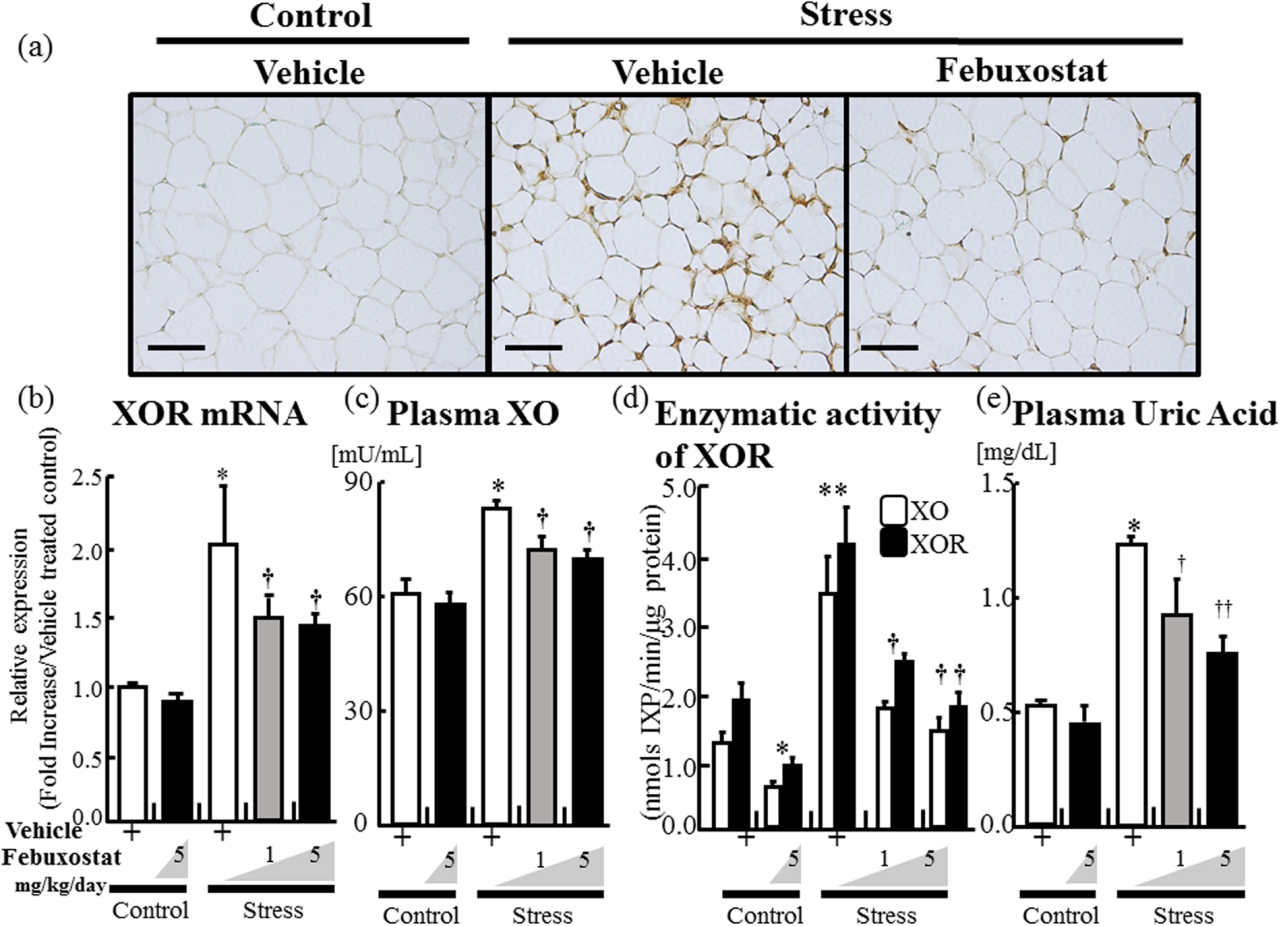
Febuxostat reduces stress-induced xanthine oxidoreductase activation and plasma uric acid levels in a restraint stress murine model. Expression levels and activities of xanthine oxidoreductase (XOR) and xanthine oxidase (XO) in adipose tissues, and plasma levels of XO and uric acid were analyzed in the control (non-stressed) and stressed mice treated with or without febuxostat (1 or 5 mg/kg/day), by immunohistochemistry, RT-PCR, ELISA, and XOR activity assay respectively. (**a**) Representative pictures of XO staining of adipose tissues (×200 magnification, bar = 50 µm). (**b**) XOR mRNA expression in adipose tissue. Data were analyzed by Student’s t-test and displayed as mean ± SD of 7 mice per group. *P < 0.001, compared with the vehicle-treated control mice, ^†^ *P* < 0.001, compared with the vehicle-treated and stressed mice. (**c**) Plasma levels of XO. Data were analyzed by Student’s t-test and displayed as mean ± SD of 7 mice per group. *P < 0.001 compared with the vehicle-treated control mice, ^†^ *P* < 0.03, compared with the vehicle-treated and stressed mice, respectively. (**d**) XO and XOR (XO + XDH) enzymatic activity in homogenized adipose tissue. Data were analyzed by Student’s t-test and displayed as mean ± SD of 5–6 mice per group. *P < 0.01 and **P < 0.001, compared with the vehicle-treated control mice, ^†^ *P* < 0.004 and ^††^ *P* < 0.003, compared with the vehicle-treated and stressed mice, respectively. (**e**) Levels of plasma uric acid. Data were analyzed by Student’s t-test and displayed as mean ± SD of 5–6 mice per group. *P < 0.01, compared with the vehicle-treated control mice, ^†^ *P* < 0.03 and ^††^ *P* < 0.012, compared with the vehicle-treated and stressed mice, respectively.

Eight-week-old male C57BL/6 J mice were randomly assigned to either the control or stress group. Control mice were left undistributed, while stressed mice were each subjected to 2 h/day of immobilization stress for two weeks, as described previously^4, 5, 17^. Immunohistochemistry and RT-PCR assay showed strong signals and increased XOR mRNA expression in VAT (inguinal adipose tissues) of stressed mice, but not in the control mice (Fig. 1a and b). Plasma XO levels were also increased in the stressed mice (Fig. 1c). Measurement of XO and XOR (XO + XDH) enzymatic activities in adipose tissue homogenates by fluorometric assay using pterin substrate^18^ showed increased XOR functional activity in stressed mice (Fig. 1d). Plasma uric acid levels were significantly higher in stressed mice than control mice (Fig. 1e). Mice of each of the two groups were divided at random into three treatment subgroups; the vehicle, 2-week treatment with 1 and 5 mg/kg/day oral febuxostat. Febuxostat significantly suppressed stress-induced increase in XOR expression and activity, and the effect was dose-dependent (Fig. 1a,b and c). The treatment resulted in marked fall in plasma uric acid level (Fig. 1d and e). The treatment also reduced the expression and activity of XOR and uric acid levels relative to the non-stressed mice (Fig. 1).

### Febuxostat suppressed free radical production in stressed mice

**Figure 2:**
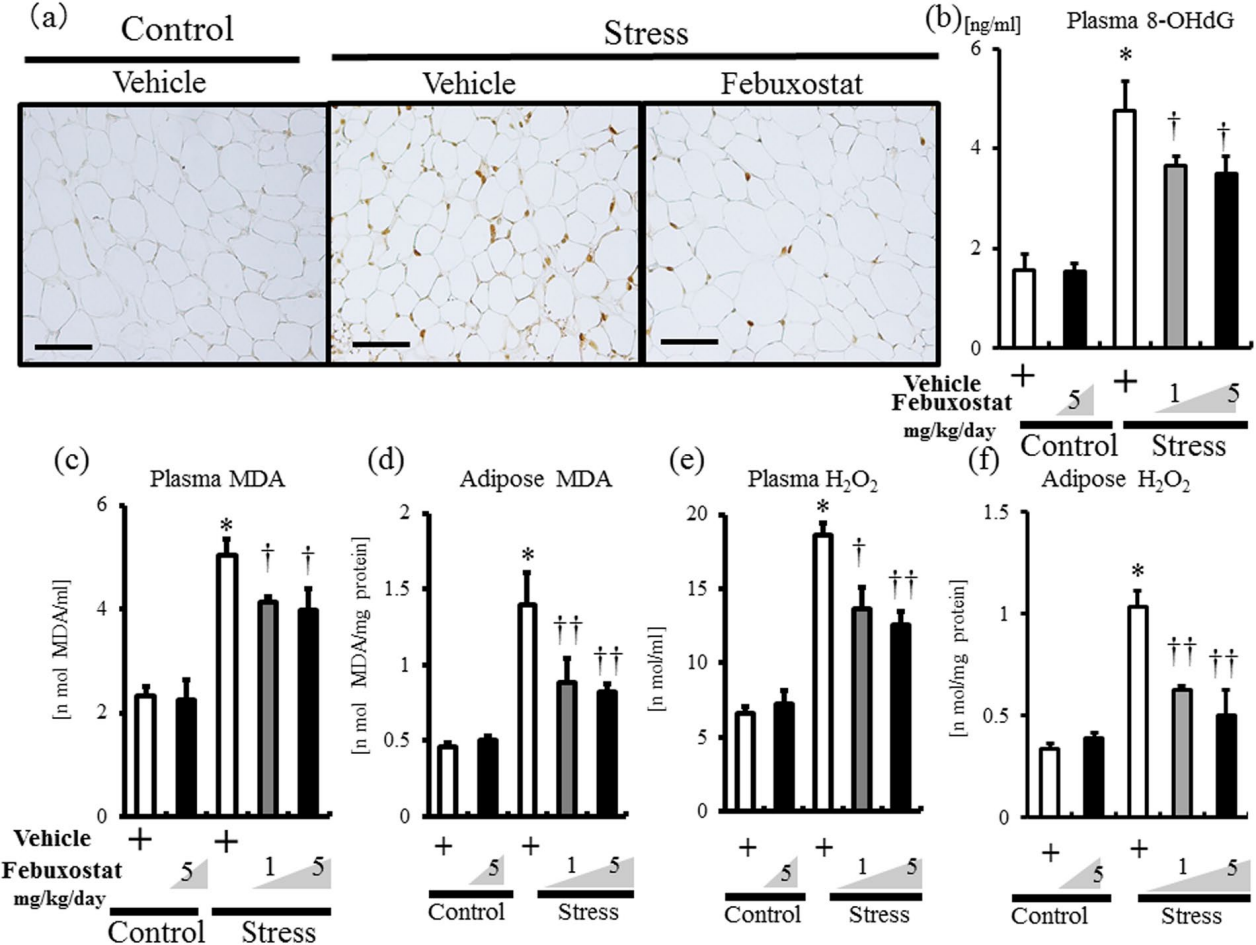
Febuxostat suppresses free radical production in stressed mice. The expression levels of 8-OHdG, lipid peroxidation (MDA) and H_2_O_2_ production were analyzed in inguinal adipose tissues and plasma of the control (non-stressed) and stressed mice treated with or without febuxostat (1 or 5 mg/kg/day) by immunohistochemistry and ELISA, respectively. Data were analyzed by Student’s t-test and displayed as mean ± SD of 5–6 mice per group. (**a**) Representative pictures of 8-OHdG staining of adipose tissues (×200 magnification, bar = 50 µm). (**b**) Plasma levels of 8-OHdG. *P < 0.001, compared with the vehicle-treated control mice, ^†^ *P* < 0.05, compared with the vehicle-treated stressed mice. (**c**,**d**) MDA in plasma (**c**) and homogenized adipose tissue (**d**). *P < 0.001, compared with the vehicle-treated control mice, ^†^ *P* < 0.05 and ^††^ *P* < 0.02, compared with the vehicle-treated stressed mice. (**e**,**f**) H_2_O_2_ in plasma (**e**) and homogenized adipose tissue (**f**). *P < 0.001, compared with the vehicle-treated control mice, ^†^ *P* < 0.002 and ^††^ *P* < 0.001, compared with the vehicle-treated stressed mice.

We measured stress-induced ROS accumulation in plasma and inguinal adipose tissues by immunohistochemistry and enzyme linked immunosorbent assays (ELISA) for 8-OHdG, a biomarker of oxidative DNA damage, malondialdehyde (MDA), which is an end-product of lipid peroxidation and a biomarker of cellular oxidative stress, and hydrogen peroxide (H_2_O_2_). As shown Fig. 2a, strong signals for 8-OHdG were recognized in inguinal adipose tissue of stressed mice. In agreement with this finding, stress markedly increased plasma 8-OHdG, and plasma and adipose tissue MDA and H_2_O_2_ (Fig. 2). Immunohistochemistry showed that the febuxostat significantly reduced 8-OHdG expression in adipose tissue of stressed mice (Fig. 2a), and also biomarkers of ROS accumulation in plasma and adipose tissue in a dose-dependent manner (Fig. 2b–f). Febuxostat treatment hardly altered the levels of these biomarkers in non-stressed mice (Fig. 2).

### Febuxostat suppressed stress-induced increase in NOX subunits

**Figure 3:**
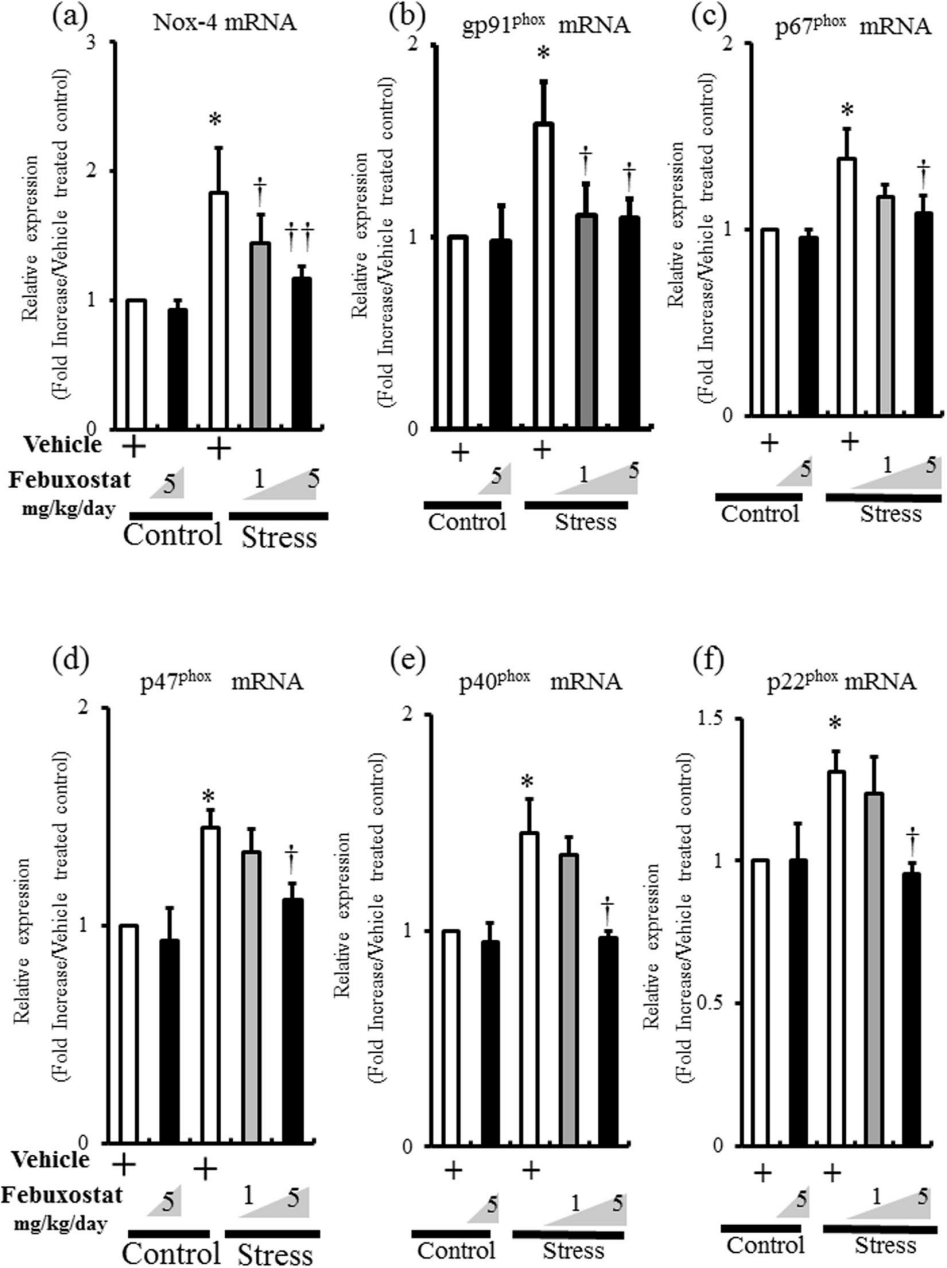
Febuxostat suppresses stress-induced increase in NOX subunits. The mRNA expression levels of NADPH oxidase subunits in inguinal adipose tissue of control mice treated with vehicle or febuxostat (5 mg/kg/day), and stressed mice treated with vehicle or febuxostat (1 or 5 mg/kg/day) were analyzed by quantitative RT-PCR. Values are expressed relative to the vehicle-treated control mice. Data were analyzed by Student’s t-test and displayed as mean ± SD of 5–6 mice per group. (**a**) NOX-4 mRNA expression in adipose tissue. *P < 0.002, compared with the vehicle-treated control mice, ^†^ *P* < 0.03 and ^††^ *P* < 0.008, compared with the vehicle-treated stressed mice. (**b**) gp91^phox^ mRNA expression in adipose tissue. *P < 0.02 vs compared with the vehicle-treated control mice, ^†^ *P* < 0.05, compared with the vehicle-treated stressed mice. (**c**) p67^phox^ mRNA expression in adipose tissue. *P < 0.02, compared with the vehicle-treated control mice, ^†^ *P* < 0.05, compared with the vehicle-treated stressed mice. (**d**) p47^phox^ mRNA expression in adipose tissue. *P < 0.002, compared with the vehicle-treated control mice, ^†^ *P* < 0.009, compared with the vehicle-treated stressed mice. (**e**) p40^phox^ mRNA expression in adipose tissue. *P < 0.004, compared with the vehicle-treated control mice, ^†^ *P* < 0.006, compared with the vehicle-treated stressed mice. (**f**) p22^phox^ mRNA expression in adipose tissue. *P < 0.017, compared with the vehicle-treated control mice, ^†^ *P* < 0.011, compared with the vehicle-treated stressed mice.

ROS accumulation in VAT is reported to be associated with increased NADPH oxidase activity^14^. Examination of the mRNA expressions of NADPH oxidase subunits in inguinal adipose tissue showed significant increase in mRNA expressions of NOX-4, gp91^phox^, p67^phox^, p47^phox^, p40^phox^ and p22^phox^ in adipose tissue of stressed mice (Fig. 3). Febuxostat did not alter the expression levels of these subunits in the non-stressed mice, but reduced stress-induced induction of NADPH oxidase subunits in a dose-dependent manner (Fig. 3).

### Febuxostat abrogated stress-induced decrease in antioxidant enzymes

**Figure 4:**
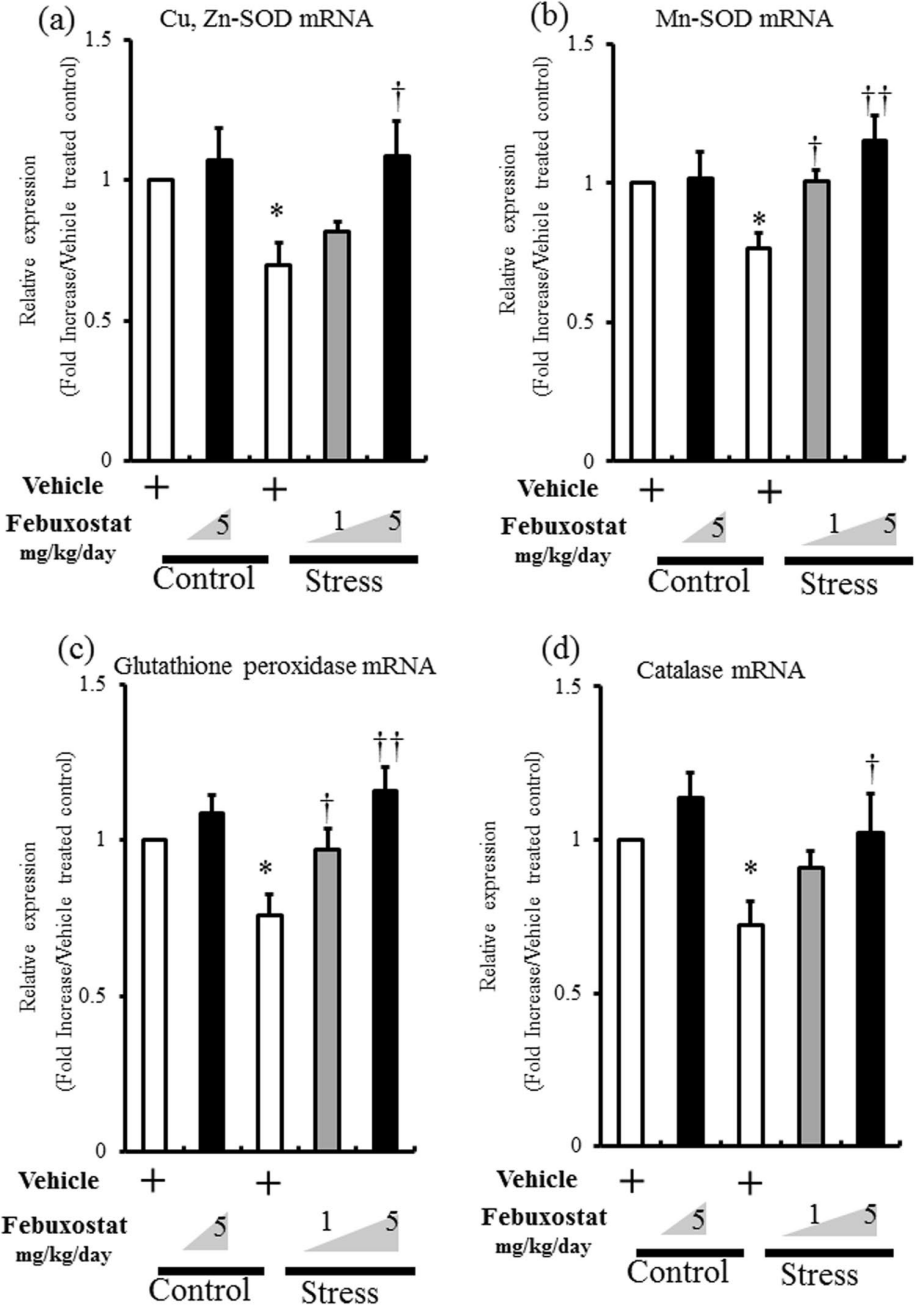
Febuxostat abrogates stress-induced decrease in antioxidant enzymes. The mRNA expression levels of antioxidant enzymes in inguinal adipose tissue of control mice treated with vehicle or febuxostat (5 mg/kg/day), and stressed mice treated with vehicle or febuxostat (1 or 5 mg/kg/day) were analyzed by quantitative RT-PCR (**a–d**). Values are expressed relative to the vehicle-treated control mice. Data were analyzed by Student’s t-test and displayed as mean ± SD of 5–6 mice per group. (**a**) Cu, Zn-SOD mRNA expression in adipose tissue. *P < 0.02, compared with the vehicle-treated control mice, ^†^ *P* < 0.04, compared with the vehicle-treated stressed mice. (**b**) Mn-SOD mRNA expression in adipose tissue. *P < 0.04, compared with the vehicle-treated control mice, ^†^ *P* < 0.04 and ^††^ *P* < 0.002, compared with the vehicle-treated stressed mice. (**c**) Glutathione peroxidase mRNA expression in adipose tissue. *P < 0.04, compared with the vehicle-treated control mice, ^†^ *P* < 0.04 and ^††^ *P* < 0.002, compared with the vehicle-treated stressed mice. (**d**) Catalase mRNA expression in adipose tissue. *P < 0.01, compared with the vehicle-treated control mice, ^†^ *P* < 0.02, compared with the vehicle-treated stressed mice.

Previous studies demonstrated the role of reduced activities of antioxidant enzymes in adipose tissue in ROS accumulation in VAT^14, 19^. Analysis of mRNA expression of antioxidant enzymes Cu, Zn-superoxide dismutase (SOD), Mn-SOD, glutathione peroxidase (GPx), and catalase in inguinal adipose tissue showed low expression levels of these antioxidant enzymes in stressed mice (Fig. 4), and that febuxostat abrogated these effects in a dose-dependent manner (Fig. 4).

### Febuxostat reduced stress-induced xanthine oxidoreductase induction in liver and intestine

**Figure 5:**
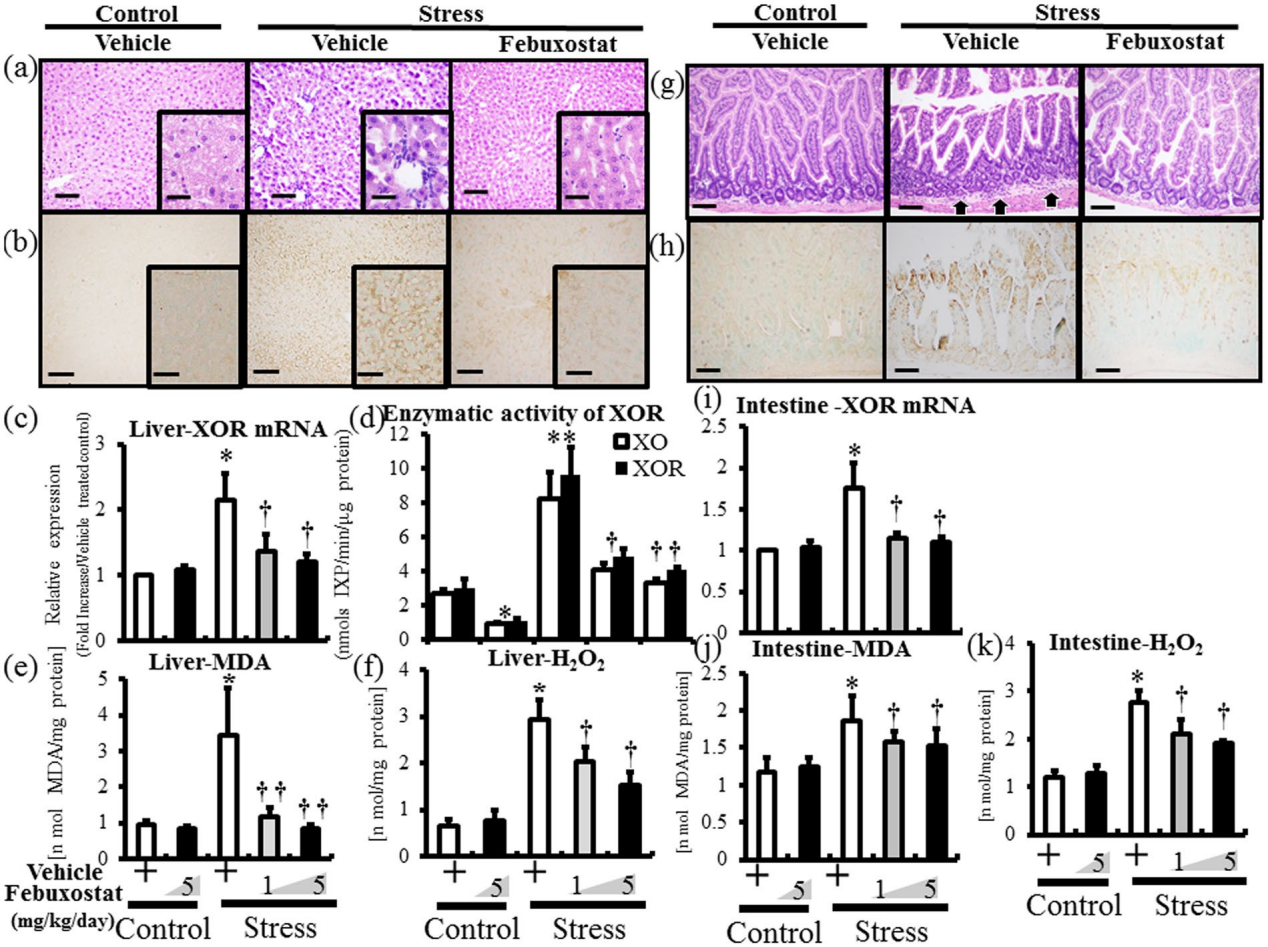
Febuxostat reduces stress-induced xanthine oxidoreductase activation and free radical production in liver and intestine. Expression levels of XOR in liver and intestine and activities of XOR in liver were analyzed in the control (non-stressed) and stressed mice treated with or without febuxostat (1 or 5 mg/kg/day), by immunohistochemistry, RT-PCR and XOR activity assay, respectively. The expression levels of MDA and H_2_O_2_ production were analyzed in homogenized liver and intestine tissues of the control (non-stressed) and stressed mice treated with or without febuxostat (1 or 5 mg/kg/day) by ELISA. Data were analyzed by Student’s t-test and displayed as mean ± SD of 5–6 mice per group. Representative pictures of HE (**a**) and XO (**b**) staining of liver (×400 magnification, bar = 25 µm; Inset, ×1000 magnification, bar = 10 µm). (**c**) XOR mRNA expression in liver. *P < 0.002, compared with the vehicle-treated control mice, ^†^ *P* < 0.05, compared with the vehicle-treated stressed mice. (**d**) XO and XOR (XO + XDH) enzymatic activity in homogenized liver tissue. *P < 0.05 and **P < 0.02, compared with the vehicle-treated control mice, ^†^ *P* < 0.03 and ^††^ *P* < 0.02, compared with the vehicle-treated and stressed mice, respectively. MDA (**e**) and H_2_O_2_ (**f**) in homogenized liver tissue. *P < 0.001, compared with the vehicle-treated control mice, ^†^ *P* < 0.05 and ^††^ *P* < 0.001, compared with the vehicle-treated stressed mice. Representative pictures of HE (**g**) and XO (**h**) staining of intestine (×200 magnification, bar = 50 µm). Arrows denotes thickening of submucosal areas with mononuclear cell infiltration. (**i**) XOR mRNA expression in intestine. *P < 0.001, compared with the vehicle-treated control mice, ^†^ *P* < 0.01, compared with the vehicle-treated stressed mice. MDA (**j**) and H_2_O_2_ (**k**) in homogenized intestine tissue. *P < 0.03, compared with the vehicle-treated control mice, ^†^ *P* < 0.05, compared with the vehicle-treated stressed mice.

Stress induced infiltration of mononuclear cells in liver, and thickening of submucosal area with mononuclear cell infiltration in intestine as previously reported^20, 21^ (Fig. 5a and g). Immunohistochemistry and RT-PCR assay showed strong signals and increased XOR mRNA expression in liver and intestine of stressed mice, compared to control mice (Fig. 5b,c,h and i). XOR and XO functional activity was also increased in liver in stressed mice (Fig. 5d). In agreement with this finding, stress markedly increased MDA and H_2_O_2_ in liver and intestine of stressed mice (Fig. 5e,f,j and k). Febuxostat abrogated these inflammatory findings and free radical production in a dose-dependent manner (Fig. 5).

### Febuxostat reduced lipolysis and FFA release in stress mice

**Figure 6:**
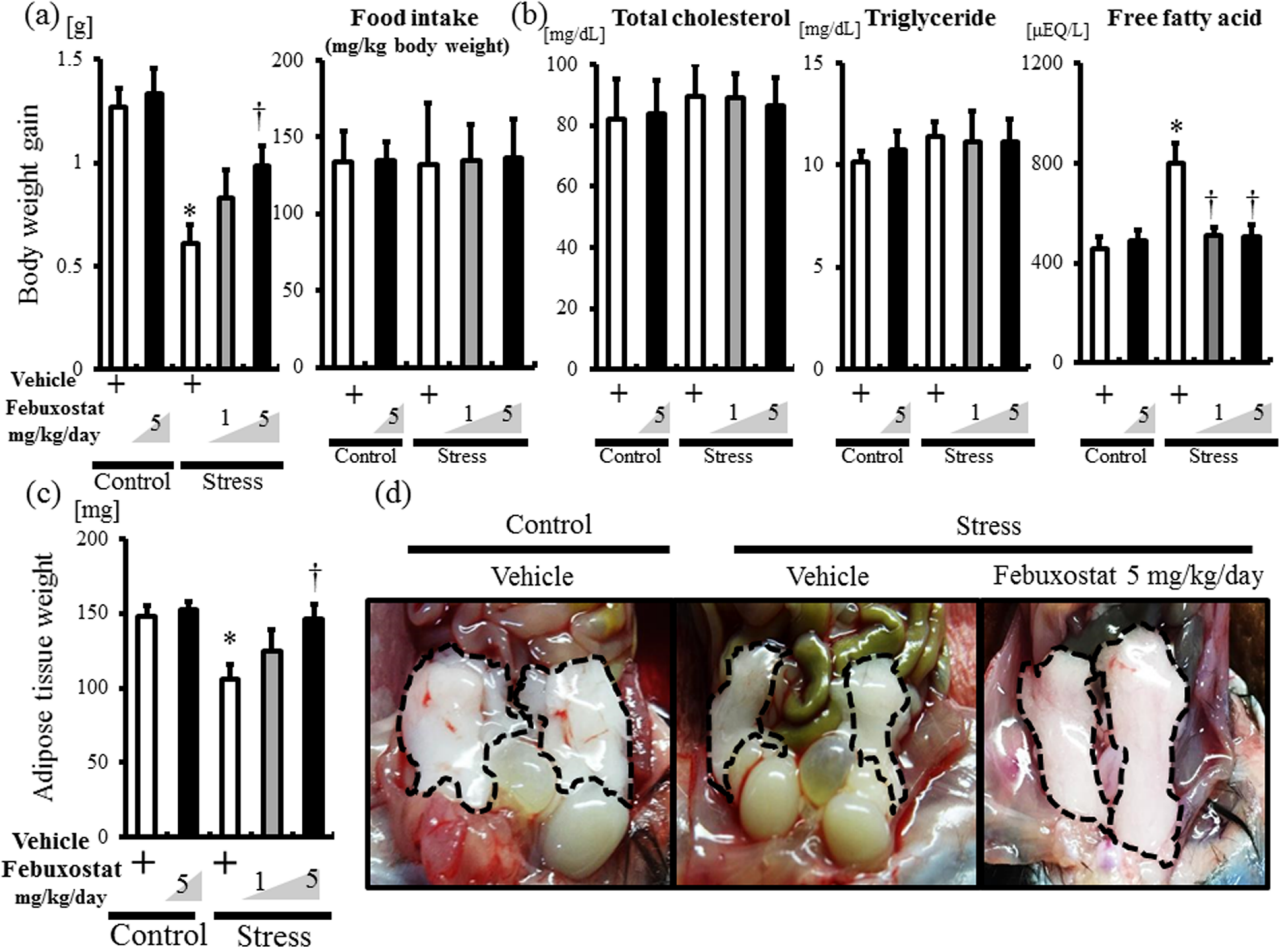
Febuxostat reduces lipolysis and free fatty acid release in stressed mice. Body weight and food intake was monitored during the stress period. At the end of the stress study, inguinal adipose tissue of each group was photographed, weighed and harvested for analysis. Data were analyzed by Student’s t-test and displayed as mean ± SD of 5–6 mice per group. (**a**) Body weight gain in control mice treated with or without febuxostat (5 mg/kg/day) and stressed mice treated with or without febuxostat (1 or 5 mg/kg/day). *P < 0.001, compared with the vehicle-treated control mice, ^†^ *P* < 0.02, compared with the vehicle-treated stressed mice. Food intake was comparable among the groups. (**b**) Plasma fat composition in the control mice treated with or without febuxostat (5 mg/kg/day) and stressed mice treated with or without febuxostat (1 or 5 mg/kg/day). Plasma levels of total cholesterol and triglyceride were comparable among the groups. Plasma free fatty acid levels. *P < 0.002, compared with the vehicle-treated control mice, ^†^ *P* < 0.005, compared with the vehicle-treated stressed mice. (**c**) Weight of inguinal adipose tissue in control mice treated with or without febuxostat (5 mg/kg/day) and stressed mice treated with or without febuxostat (1 or 5 mg/kg/day). *P < 0.003, compared with the vehicle-treated control mice, ^†^ *P* < 0.008 compared with the vehicle-treated stressed mice. (**d**) Representative pictures of inguinal fat pad in control (non-treated), stressed and febuxostat (5 mg/kg/day) treated mice. Circle dot line: adipose tissue.

Body weight was weighed during the 2-week stress period. Body weight gain was significantly reduced in stressed mice compared to the non-stressed control (Fig. 6a), and treatment with 5 mg/kg/day febuxostat, but not 1 mg/kg/day, abrogated the effect of stress on body weight (Fig. 6a). However, high-dose febuxostat did not alter body weight gain of non-stressed mice (Fig. 6a). Each group of mice consumed almost similar amounts of food (approximately 130 mg/g/day; Fig. 6a).

Analysis of lipid composition showed comparable changes in total cholesterol and triglyceride levels in the different treatment groups (Fig. 6b). However, plasma FFA levels were significantly higher in stressed mice (Fig. 6b). Furthermore, febuxostat reduced FFA levels in a dose-dependent manner (Fig. 6b). Inguinal adipose tissue weight was significantly lower in stressed mice than non-stressed mice, and this decrease was abrogated by high-dose febuxostat (Fig. 6c). Gross examination of VAT showed shrinkage of inguinal fat pad of stressed mice, and this effect was abrogated by high-dose febuxostat (Fig. 6d). These results indicate that febuxostat reduces stress-induced lipolysis and FFA release.

### Febuxostat prevented adipose tissue inflammation in stress mice

**Figure 7:**
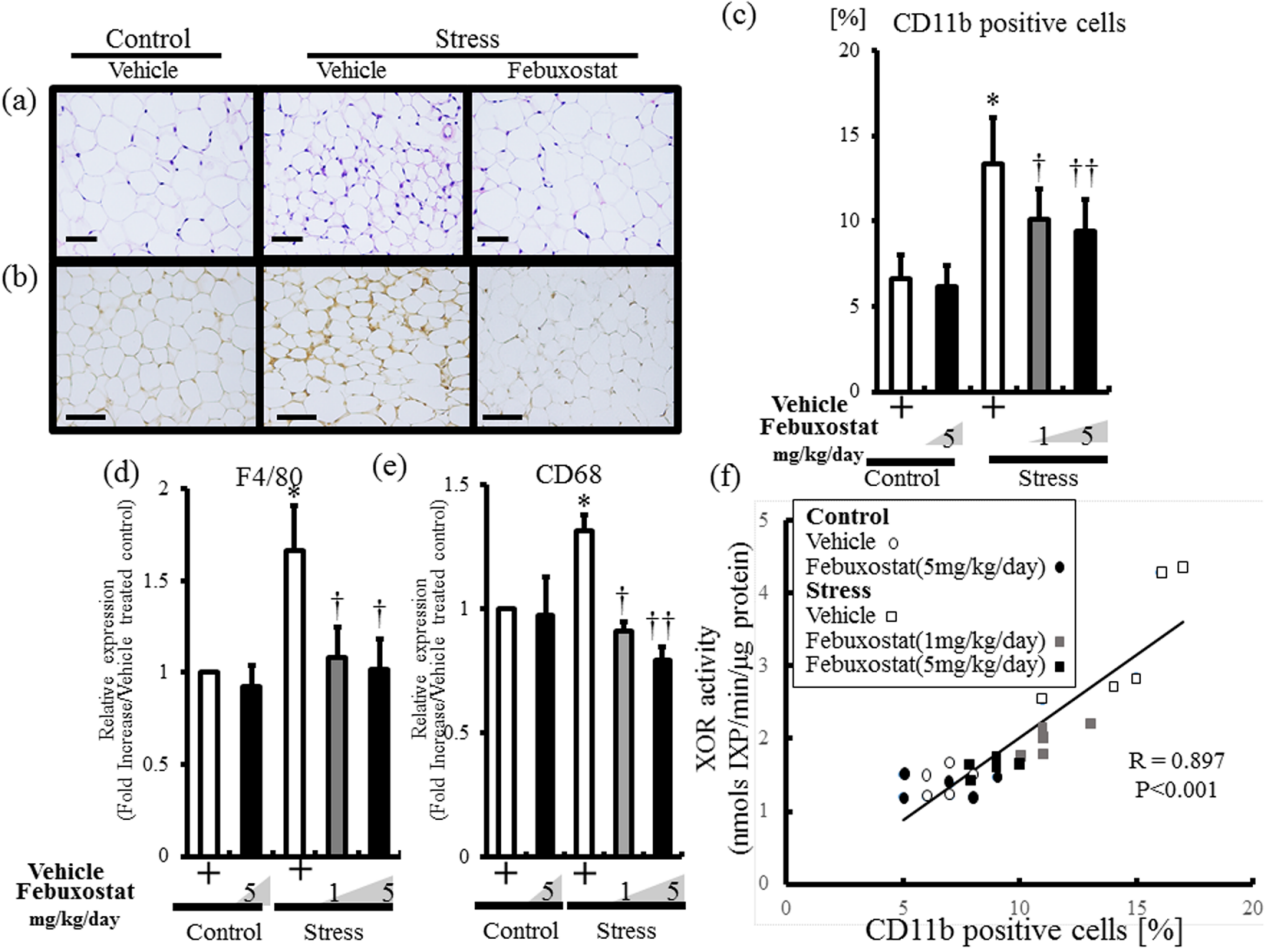
Febuxostat prevents stress-induced adipose tissue inflammation. Inguinal adipose tissues of stressed and control (non-stressed) mice were analyzed by H & E staining (**a**), CD11b immunostaining (**b**,**c**) and mRNA expression levels of CD68 and F4/80 (**d**,**e**). (**a**) Accumulation of mononuclear cells in inguinal adipose tissues following 2-week restraint stress. Top panel, ×200 magnification, bar = 50 µm. (**b**) CD11b-positive cells (monocytes) in adipose tissue of stressed mice (×200 magnification, bar = 50 µm). (**c**) Quantitative analysis of CD11b-positive cells relative to total number of nuclei. Data are mean ± SD of 8 mice per group. *P < 0.001, compared with the vehicle-treated control mice, ^†^ *P* < 0.002 and ^††^ *P* < 0.001, compared with the vehicle-treated stressed mice. (**d**) and (**e**) Quantitative analysis of F4/80 (**d**) and CD68 (**e**) expression levels in adipose tissue. Values are expressed relative to the vehicle-treated control mice. Data were analyzed by Student’s t-test and displayed as mean ± SD of 5 mice per group. (**d**) Quantitative analysis of F4/80 mRNA expression in adipose tissue. *P < 0.001, compared with the vehicle-treated control mice, ^†^ *P* < 0.001, compared with the vehicle-treated stressed mice. (**e**) Quantitative analysis of CD68 mRNA expression in adipose tissue. *P < 0.02, compared with the vehicle-treated control mice, ^†^ *P* < 0.002 and ^††^ *P* < 0.001, compared with the vehicle-treated stressed mice. (**f**) The relationship between XOR activity and CD11b positive cells was analyzed by Pearson’s correlation coefficient. XOR activity correlated significantly with CD11b positive cells (*P* < 0.001, R = 0.897).

As reported previously^4^, significant accumulation of mononuclear cells was observed in VAT of stressed and vehicle-treated mice compared to non-stressed mice (Fig. 7a). Analysis of expression of macrophage surface markers demonstrated that stress was associated with a significant increase in CD11b-positive cells and upregulation of monocyte/macrophage cell surface markers (F4/80 and CD68) in WAT (Fig. 7b–e). Febuxostat markedly reduced monocyte accumulation and mRNA expression levels of monocyte surface markers in WAT of stressed mice in a dose-dependent manner. The high dose of febuxostat did not alter monocyte accumulation in control mice. There were significant correlation between XOR activity and monocyte accumulation in VAT (Fig. 7f). XOR activity and monocyte accumulation were similarly increased by stress, and were decreased by febuxostat in a dose-dependent manner.

### Febuxostat reduced inflammatory adipokine levels in stress mice

**Figure 8:**
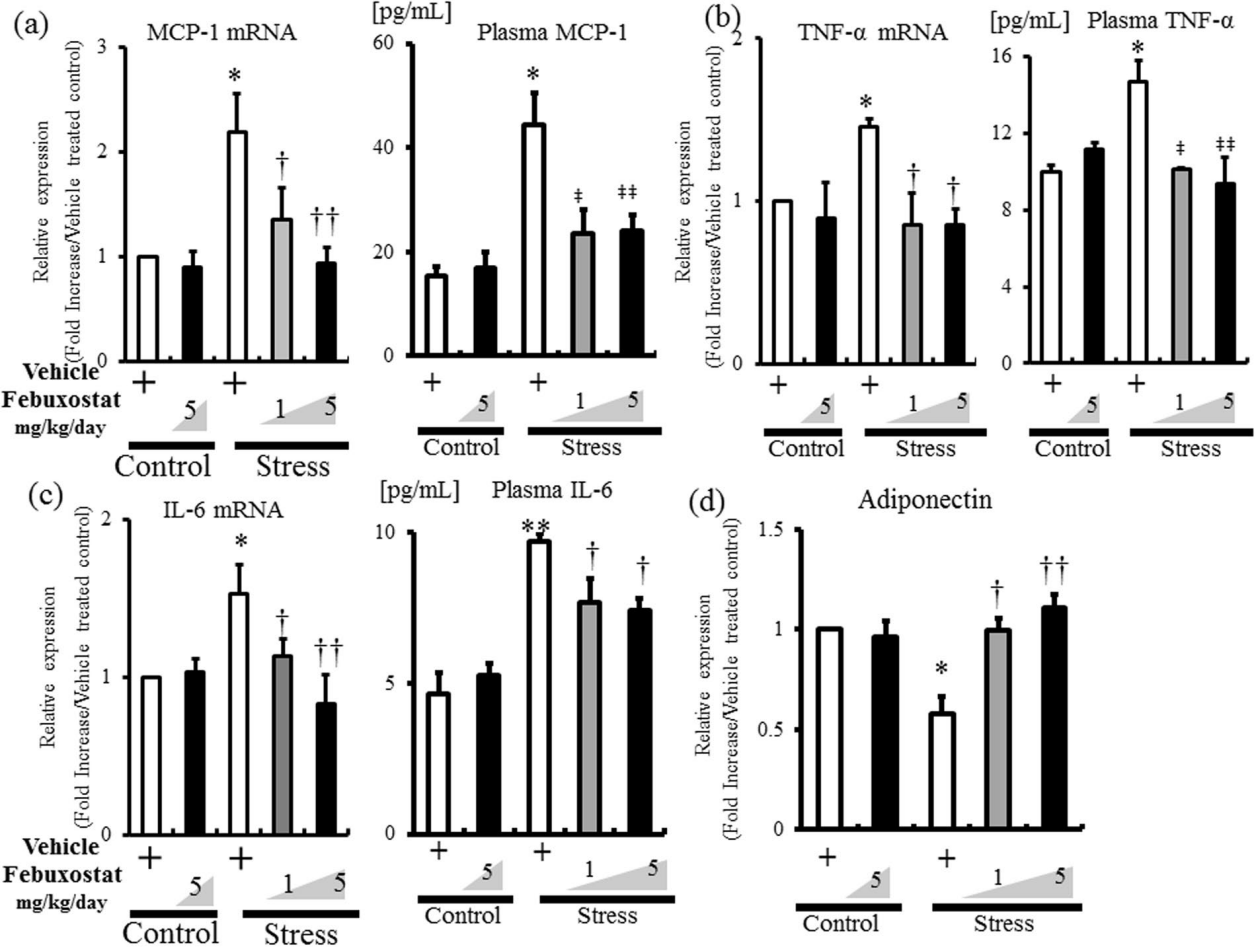
Febuxostat reduces the expression of stress-induced proinflammatory adipokines and restores adiponectin expression in adipose tissue. The mRNA expression levels of MCP-1 (**a**), TNF-α (**b**), IL-6 (**c**), and adiponectin (**d**) in inguinal adipose tissues of control mice treated with vehicle or febuxostat (5 mg/kg/day), and stressed mice treated with vehicle or febuxostat (1 or 5 mg/kg/day) were analyzed by quantitative RT-PCR. Values are expressed relative to the vehicle-treated control mice. Plasma concentrations of MCP-1, TNF-α and IL-6 (**a**–**c**), respectively) were also measured in the same groups. Data were analyzed by Student’s t-test and displayed as mean ± SD of 5–6 mice for RT-PCR and ELISA per group. (**a**) *P < 0.001, compared with the vehicle-treated control mice, ^†^ *P* < 0.03, ^††^ *P* < 0.001, ^‡^ *P* < 0.003 and ^‡‡^ *P* < 0.002, compared with the vehicle-treated stressed mice, respectively. (**b**) *P < 0.01, compared with the vehicle-treated control mice, ^†^ *P* < 0.001, ^‡^ *P* < 0.02 and ^‡‡^ *P* < 0.002, compared with the vehicle-treated stressed mice, respectively. (**c**) *P < 0.005 and **P < 0.001, compared with the vehicle-treated control mice, ^†^ *P* < 0.02, and ^††^ *P* < 0.001, compared with the vehicle-treated stressed mice, respectively. (**d**) *P < 0.005, compared with the vehicle-treated control mice, ^†^ *P* < 0.02, and ^††^ *P* < 0.01, compared with the vehicle-treated stressed mice, respectively.

We reported in previous studies^4, 8, 9^ that restraint stress induced proinflammatory adipokines in VATs. The 2-week restraint stress applied in the present study also resulted in upregulation of MCP-1, TNF-α, and IL-6 expression in VATs, and these changes were suppressed in a dose-dependent manner by febuxostat (Fig. 8a–c). Febuxostat treatment also reduced the elevated levels of plasma MCP-1, TNF-α, and IL-6 in stressed mice, in parallel with the changes in their mRNA expression levels in VATs (Fig. 8a–c). The anti-inflammatory adipokine, adiponectin, was significantly downregulated in VATs of stressed mice compared to the control mice, as reported previously^4^. Febuxostat treatment abrogated this effect dose-dependently (Fig. 8d). However, there were no significant changes in the expression levels of these adipokines in the WAT of control mice treated with vehicle and with higher-dose febuxostat.

### Febuxostat improved stress-induced insulin sensitivity and prothrombotic state

**Figure 9:**
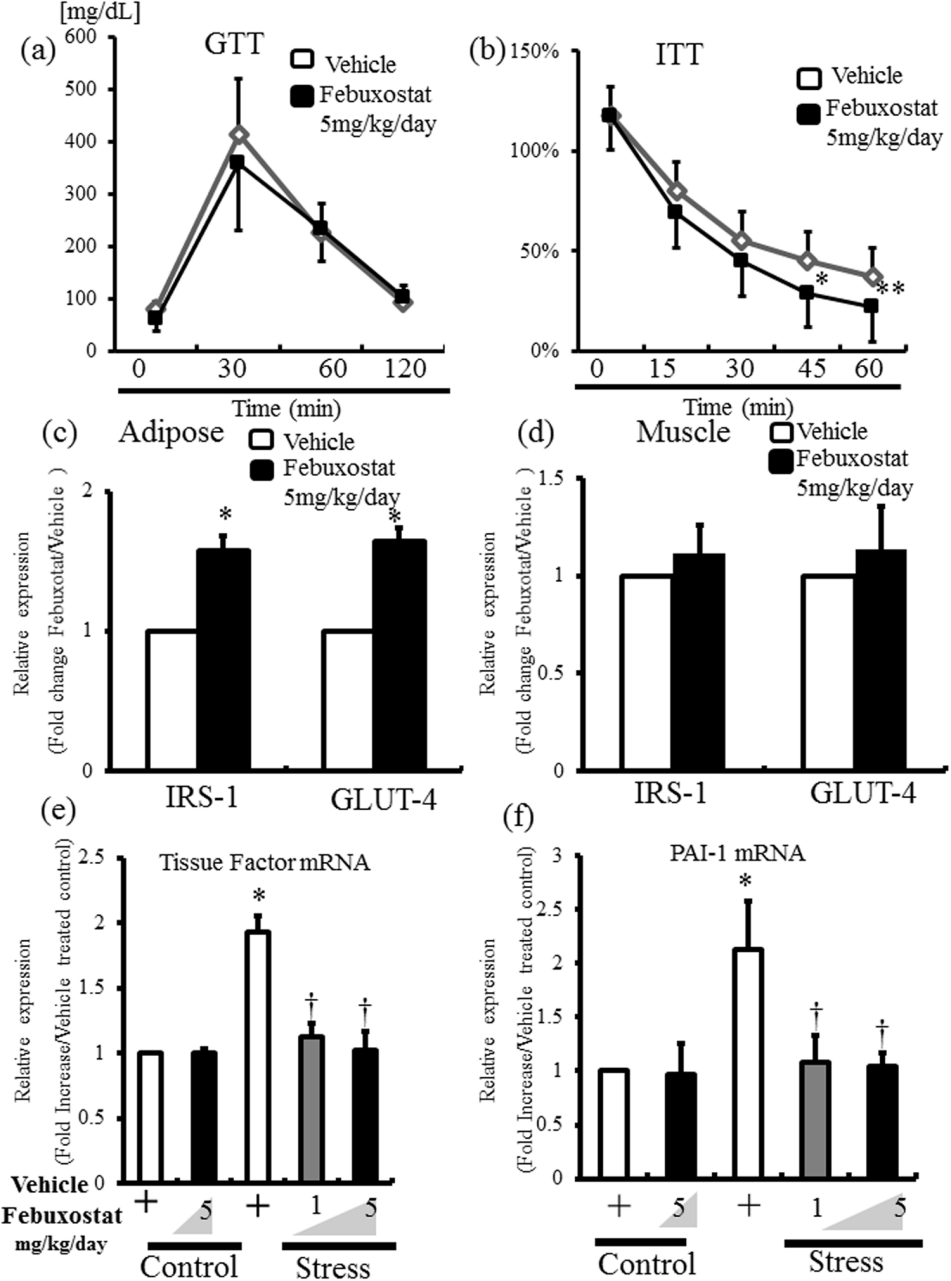
Febuxostat improves stress-induced insulin resistance and prothrombotic state. After two weeks of daily stress, intraperitoneal glucose tolerance (GTT) and insulin tolerance tests (ITT) were performed in the vehicle- and febuxostat- (5 mg/kg/day) treated mice. The mRNA expression levels of IRS-1 and GLUT4, in inguinal adipose tissue and skeletal muscle (adductor muscle), and PAI-1 and tissue factor in inguinal adipose tissue were analyzed by quantitative RT-PCR. Data were analyzed by Student’s t-test and displayed as mean ± SD of 8 mice per group. (**a**) Glucose tolerance was comparable between the stressed mice treated with vehicle and febuxostat after stress. (**b**) Insulin tolerance showed significant recovery in febuxostat-treated and stressed mice. *P < 0.05, and **P < 0.04, compared with the vehicle-treated and stressed mice. (**c**,**d**) Quantitative analysis of IRS-1 and GLUT4 expression levels in inguinal adipose tissue (**c**) and skeletal muscle (adductor muscle) (**d**) of stressed mice treated with vehicle or febuxostat (5 mg/kg/day). *P < 0.001, compared with vehicle-treated and stressed mice. (**e**,**f**) Quantitative analysis of tissue factor and PAI-1 expression in inguinal adipose tissue of the control and stressed mice treated with vehicle or febuxostat (1 or 5 mg/kg/day). *P < 0.001, compared with vehicle-treated control mice, ^†^ *P* < 0.001, compared with vehicle-treated stressed mice.

We reported recently that 2-week restraint stress reduced insulin sensitivity downstream of low-grade adipose inflammation, and that anti-inflammatory therapy restored insulin resistance^4, 8^. In this study, glucose tolerance test (GTT) and insulin tolerance test (ITT) were conducted in unstressed and stressed mice with the vehicle- or febuxostat-treatment (5 mg/kg/day). In these tests, fasting was imposed overnight in GTT and for 16 hours in ITT. There was no significant difference in glucose tolerance between unstressed and stressed mice with the vehicle treatment (data not shown). In both unstressed and stressed mice, the febuxostat-treatment did not alter glucose tolerance (Fig. 9a and Supplemental Figure 1a). Stress significantly impaired insulin sensitivity after 45 min in the vehicle treated mice (unstressed mice vs stressed mice; 30.6 ± 5.6% vs 47.4% ± 16 at 45 min, n = 8, p < 0.05; 23.4 ± 3.3% vs 36.6 ± 14% at 60 min, n = 8, p < 0.006). On the other hand, febuxostat at 5 mg/kg/day significantly improved insulin tolerance in stressed mice at 45 min (Fig. 9b). The febuxostat treatment did not alter insulin tolerance in unstressed mice (Supplemental Figure 1b).

Finally, the mRNA expression levels of glucose transporter (GLUT-4) and insulin receptor substrate (IRS-1) in adipose tissue and skeletal muscles (adductor muscles) was analyzed in unstressed and stressed mice. The febuxostat treatment did not alter GLUT-4 and IRS-1 expression in adipose and skeletal muscles in unstressed mice (Supplemental Figure 1c and d). Stress significantly reduced GLUT-4 and IRS-1 expression in VAT in vehicle-treated mice (−22.0 ± 9.7% in GLUT-4, n = 8, p < 0.004; −18.0 ± 9.8% in IRS-1, n = 8, p < 0.004). Meanwhile, significant upregulation of both molecules was observed in VAT of the stressed mice treated with febuxostat (5 mg/kg/day), compared to the vehicle-treated mice (Fig. 9c), though no such effects were noted in the skeletal muscle (Fig. 9d). Restraint stress has also been demonstrated to induce the expression of prothrombotic factors (PAI-1 and TF) in VAT^5^. PAI-1 and TF were significantly upregulated in inguinal adipose tissue of stressed mice but not in non-stressed mice, and these effects were abrogated by febuxostat in a dose dependent manner (Fig. 9e,f).

Considered together, the above findings indicate that febuxostat suppresses stress-induced rise in plasma uric acid, lipolysis and adipose tissue inflammation and improves glucose metabolism and prothrombotic state.

## Discussion

The novel findings of this study was that chronic stress-induced VAT inflammation, hyperuricemia, disorders of glucose metabolism and prothrombotic state, were all abrogated by administration with febuxostat, and the effects were dose-dependent. The results showed that 2-week intermittent restraint stress induced simultaneous increase in plasma uric acid levels and ROS generation downstream of XOR activation in VAT, liver and intestine (Figs 1, 2 and 5). The stress-induced ROS generation augmented NOX subunits and reduced antioxidant enzyme activities in VAT (Figs 3 and 4). In addition, stress induced lipolysis (Fig. 6) and adipose tissue inflammation (Figs 7 and 8), decreased insulin sensitivity, and prothrombotic state (Fig. 9), confirming the results of previous studies^4^. Thus, febuxostat suppressed stress-induced lipolysis, adipose inflammation and ROS production, resulting in restoration of glucose and uric acid metabolism, and reduced thrombotic tendency.

Growing evidence shows that hyperuricemia is an independent risk factor for cardiovascular and renal diseases, and plays an important role in many disease states such as gout, articular degenerative disorders, chronic kidney disease and atherosclerosis^22, 23^. The relationship between psychological stress and serum levels of uric acid has been discussed due to its clinical significance^1^, but there is no information on how stress affects uric acid metabolism. Uric acid level is regulated by a balance between uric acid formation and excretion driven by several enzymatic pathways, including XOR. XOR mRNA expression has been detected in most tissues, and the highest transcript levels and its activity were found in liver and intestine^15^. Adipose tissue is another important source of XOR in mice in special condition such as obesity^12^ and diabetes^15^. In the present study, stress induced XOR expression and activity in liver, intestine and adipose (Figs 1 and 5) in accordance with inflammatory findings (Figs 5 and 7f). Stressor exposure increases the synthesis and release of inflammatory proteins both in the blood and within tissues such as liver to evoke sterile inflammation^20^. We also reported that chronic restraint stress evokes low-grade chronic inflammation in the adipose tissue^4^. Circulating the inflammatory cytokines such as interleukin-1, IL-6, TNF-α would induce XOR expression and activation, at least in these organs, to upregulate production of uric acid^13^. Typically, serum uric acid levels are low in mice since uric acid is converted into allantoin by uricase^11^. Nevertheless, the 2-week intermittent restraint stress markedly increased XOR expression and activity in VAT as well as liver to increase uric acid levels (Fig. 1). In stressed subjects, stress-induced cortisol release and adipose SNS activation initiate lipolysis (Fig. 6)^4^. The process of lipolysis is involved in the turnover of cAMP, which is the intracellular messenger of catecholamines^24^. Hydrolysis of cAMP yields AMP, which is the first substrate of the catabolic reaction of purine production. Therefore, lipolysis seems also related to uric acid production in stressed subjects^12^. We demonstrated recently that stress as well as Mets evoked chronic low-grade inflammation downstream of MCP-1 production and reduced adiponectin in VAT, and identified VAT as a major target organ for stress^4^.

Uric acid also stimulates adipocytes to secrete proinflammatory adipokines, including MCP-1, and reduce anti-inflammatory adipokine, adiponectin^25^. Meanwhile, XOR inhibitor, allopurinol, improved the proinflammatory endocrine imbalance in the adipose tissue by reducing the production of MCP-1 and increasing the production of adiponectin^25^. In the present study, XOR specific inhibitor, febuxostat, suppressed stress-induced production of proinflammatory adipokine and ROS, and restored adiponectin expression in VAT (Fig. 8). Furthermore, febuxostat also suppressed stress-induced lipolysis in accordance with the decrease in VAT inflammation (Figs 4, 6 and 7). Macrophage-derived TNF-α, which is derived from infiltrated macrophages and adipocytes in WAT, acts on TNF-α receptor in hypertrophied adipocytes, thereby inducing proinflammatory cytokine production and adipocyte lipolysis via NF-κB and MAPK-dependent mechanisms, respectively^26^. In the present study, febuxostat reduced monocyte accumulation and TNF-α induction, and thus broke the vicious circle between stress-induced lipolysis and adipose tissue inflammation (Figs 6–8). Thus, both uric acid and XOR coordinately play critical role in uric acid metabolism and VAT inflammation under stress conditions.

We demonstrated recently that chronic stress increased ROS accumulation in VAT downstream of the renin-angiotensin system (RAS) activation^8, 9^. Furthermore, we also showed here that the stress procedure upregulated the subunits of NADPH oxidase, which is a major source of ROS in adipocytes, and downregulated antioxidant enzymes in VAT (Figs 3 and 4). Obesity-induced ROS production in VAT is involved in quite similar fashion. In VAT of Mets animals, the expression levels of NADPH oxidase subunits was increased downstream of induction of PU.1, which is known to increase the transcription of NADPH oxidase subunits in myeloid cells, and decreased those of antioxidant enzymes^14^. The mechanisms responsible for changes in the expression levels of NADPH oxidase subunits and antioxidant enzymes in VAT in the presence of low grade inflammation remain elusive at present, but the behavior of these molecules under stress was quite similar to that in Mets. In addition to activation of RAS, FFA also activate NADPH oxidase in adipocytes^14^. Stress-induced lipolysis and FFA release would be involved also in NADPH activation (Figs 3 and 4)^4^. Thus, stress seems to stimulate VAT to produce ROS in a coordinated manner to modulate NADPH oxidase activation. Both ROS generation and inflammatory adipokines in adipose tissue synergistically induced PAI-1 and TF in adipose tissue (Fig. 9)^27^. XO activation increased the expression levels of PAI-1 and TF to exacerbate pro-thrombotic state via ROS accumulation, in a manner similar to that in Mets animal model^14, 28^. It has been reported that febuxostat inhibits endothelial XO to reduce ROS production, and preserves the activity of tissue factor pathway inhibitor to bind and inactivate factor Xa^29^. Thus, febuxostat has antithrombotic properties, which act to reduce stress-induced ROS generation and inflammatory adipokine production.

We reported previously that stress-induced adipose tissue inflammation resulted in low insulin sensitivity and downregulation of IRS-1 and GLUT4 in VAT^4^ and that treatment with anti-inflammatory agents like angiotensin II receptor blocker and dipeptidyl peptidase-4 inhibitor, suppressed VAT inflammation and restored IRS-1 and GLUT4 expression in VAT, as well as insulin sensitivity^8, 9^. In the present study, febuxostat restored the expression levels of IRS-1 and GLUT4 in VAT, and insulin sensitivity in parallel with a decrease in ROS accumulation and inflammation in VAT (Figs 7–9). While there are still questions on whether ROS directly trigger insulin resistance in adipocytes^30^, it is reported that ROS activates NF-κB signaling to induce pro-inflammatory adipokines, such as MCP-1, TNF-α, and IL-6^31^. Febuxostat treatment restored IRS-1 and GLUT4 expression in VAT through significant suppression of adipose TNF-α^32^. Furthermore, the anti-inflammatory effect of febuxostat should improve insulin resistance because any decrease in plasma IL-6 is also expected to functionally improve insulin signaling in skeletal muscles at IRS-1 function, which is independent of IRS-1 and GLUT4 expression^33^, and restoration of adiponectin levels in VAT would also help in the recovery of systemic insulin sensitivity^34^.

There are substantial differences in uric acid metabolic process between human and mice. As shown above, uric acid is rapidly converted into allantoin by uricase in the mice but not in the human^11^. The activity and organ distribution of XOR differs between human and mice^15, 35^. In general, basal expression and activity of XOR in the human tissue is significantly lower compared to the mice. The expression level and activity of XOR in adipose is also low in the human^15, 35^. These would make it difficult to extrapolate our present study to human. But there is compelling evidence to support an aetiological role for inflammation and oxidative stress in the pathophysiology of mental stress^36^. XOR would plays a critical role in ROS production and alter uric acid metabolism in the downstream of inflammatory reaction in human under stressful condition.

In conclusion, our findings suggest that febuxostat inhibits stress-induced XOR activation to suppress VAT inflammation, and rectifies disorders of uric acid and glucose metabolism, and prothrombotic state. XO inhibition by febuxostat might be a potential therapy for stress-related disorders.

## Methods

### Experimental animals

Eight-week-old male C57BL/6 J mice (Chubu Kagaku Shizai Co, Nagoya, Japan) were housed two per cage under standard conditions (23 ± 1 °C, 50 ± 5% humidity), with 12-h light/dark cycle (lights on at 7:30 a.m.) in a viral pathogen-free facility at the Division for Research of Laboratory Animals, Nagoya University Graduate School of Medicine^4, 5^. The study protocol was approved by the Institutional Animal Care and Use Committee of Nagoya University (Protocol Number 27009), and the study was performed according to the Guide for the Care and Use of Laboratory Animals published by the National Institutes of Health.

### Restraint stress procedure

Mice were randomly divided into the control (n = 16) and stress groups (n = 25). Control mice were undisturbed and allowed contact with each other, while stressed mice were kept in individual cages and subjected to two hour per day of immobilization stress over a period of two weeks as we described in a previous study^4^. Briefly, within each group, mice were randomly assigned to either CE-2 diet mixed with the vehicle (0.4% methylcellulose) or febuxostat (1 or 5 mg/kg/day, a kind gift from Teijin Pharma Ltd., Tokyo, Japan), 8–9 mice per group, for two weeks. The control animals received vehicle or 5 mg/kg/day febuxostat. The stressed animals were given vehicle, or low dose (1 mg/kg/day) or high dose (5 mg/kg/day) febuxostat. Body weight and food intake were monitored/weighed in every 2 days during the stress period (between 10 am and 12 noon, 6 days a week). Animals were anesthetized with 150 mg/kg sodium pentobarbital intraperitoneally and euthanized in the morning next to the last restraint stress. Biological samples were collected for total RNA extraction, analysis of plasma lipid profile, expression levels of biological markers, and pathological examination^37^. Plasma uric acid, total cholesterol, triglyceride, and free fatty acid (FFA) levels were measured with standard enzymatic methods or ELISA using Hitachi LABOSPECT 008 (Hitachi High-Technologies Corporation, Tokyo).

### Quantitative PCR

Total RNA extraction, reverse-transcription, and quantitative PCR were performed as described previously^37^. Briefly, total RNA from the adipose, liver and intestine was extracted using TRIzol reagent (Life Technologies, Carlsbad, CA) according to the instructions provided by the manufacturer, and converted to cDNA using SuperScript VILO MasterMix (Life Technologies). The cDNA was amplified using SYBRH Green (Life Technologies) with gene-specific primers on ABI PRISM 7500 system (Life Technologies). The oligonucleotide primers used in the experiments are listed in Table 1. The amount of each RNA was normalized to the respective β-actin mRNA.

**Table 1:** Sequences of primers used for RT-PCR.

Gene	Forward (5′–3′)	Reverse (5′–3′)	length
XOR	TATGACCGCCTTCAGAAC	TATGCCTTCCACAGTTGT	102
NOX4	ACTCCTTGGGTCAGCACTGG	GTTCCTGTCCAGTTGTCTTCG	160
gp91^phox^	CAAGATGGAGGTGGGACAGT	GCTTATCACAGCCACAAGCA	170
p67^phox^	CTGGCTGAGGCCATCAGACT	AGGCCACTGCAGAGTGCTTG	214
p47^phox^	GATGTTCCCCATTGAGGCCG	GTTTCAGGTCATCAGGCCGC	212
p40^phox^	GCCGCTATCGCCAGTTCTAC	GCAGGCTCAGGAGGTTCTTC	189
p22^phox^	GTCCACCATGGAGCGATGTG	CAATGGCCAAGCAGACGGTC	164
Cu,Zn-SOD	CAGCATGGGTTCCACGTCCA	CACATTGGCCACACCGTCCT	168
Mn-SOD	CACATTAACGCGCAGATCATG	CCAGAGCCTCGTGGTACTTCTC	100
Glutathione peroxidase	GGGCAAGGTGCTGCTCATTG	AGAGCGGGTGAGCCTTCTCA	269
Catalase	CCAGCGACCAGATGAAGCAG	CCACTCTCTCAGGAATCCGC	198
F4/80	CTTTGGCTATGGGCTTCCAGTC	GCAAGGAGGACAGAGTTTATCGTG	165
CD68	ACTTCGGGCCATGTTTCTCT	GGCTGGTAGGTTGATTGTCGT	139
MCP-1	TCAGCCAGATGCAGTTAACGC	TGATCCTCTTGTAGCTCTCCAGC	95
TNF-α	AGGCTGCCCCGACTACGT	GACTTTCTCCTGGTATGAGATAGCAAA	70
IL-6	CCAGAGATACAAAGAAATGATGG	ACTCCAGAAGACCAGAGGAAAT	88
Adiponectin	GCCGCTTATGTGTATCGCTCAG	GCCAGTGCTGCCGTCATAATG	126
IRS-1	GTGAACCTCAGTCCCAACCATAAC	CCGGCACCCTTGAGTGTCT	66
GLUT-4	CAGCTCTCAGGCATCAAT	TCTACTAAGAGCACCGAG	140
Tissue Factor (TF)	TCAAGCACGGGAAAGAAAAC	CTGCTTCCTGGGCTATTTTG	137
PAI-1	ACAGCCTTTGTCATCTCAGCC	CCGAACCACAAAGAGAAAGGA	75
β-Actin	TATTGGCAACGAGCGGTTC	ATGCCACAGGATTCCATACCC	75

### Histological analysis

Inguinal adipose tissue (VAT), liver and intestine samples were harvested after euthanasia and subjected to immunohistochemistry, using the streptavidin-biotinylated peroxidase complex method. The adipose tissue was immunohistochemical stained for CD11b (1:100; Abcam Inc., Cambridge, MA), XO (1:100, Abcam) and 8-OHdG (1 µg/ml; Japan Institute for the Control of Aging, Fukuroi, Japan) using standard protocols as described previously^8, 9^. Two investigators blindly and independently counted the number of CD11b- and 8OHdG-positive and -negative cells under a microscope at ×200 magnification. Ten microscopic fields were chosen in three different sections per mouse for examination.

### Biochemical measurements

Plasma cytokine levels were quantified using mouse CCL2 ELISA Ready-SET-Go (eBioscience, Kobe, Japan), and TNF-α and IL-6 ELISA kit (R&D Systems, Minneapolis, MN) according to the instructions provided by the respective manufacturers^4, 9^. Plasma 8-OHdG levels were determined with a competitive ELISA kit (8-OHdG Check, Highly Sensitive kit, Japan Institute for the Control of Aging)^9^.

### Xanthine oxidase (XO) and xanthine oxidoreductase (XOR) activity assay

XO and XOR activities in adipose and liver tissue homogenates were measured according to pterin-based assay as reported previously^18, 38^. Briefly, inguinal adipose tissue was homogenized in 50 mM phosphate buffer (pH 7.4) containing 1 mM EDTA and protease inhibitor cocktail. Samples were centrifuged at 15,000 × g for 15 min at 4 °C. Then, adipose samples were reacted with 20 μL of 1 mM of pterin to measure XO activity. Subsequently, 20 μL of 1 mM methylene blue was further added to the tissue samples to measure total XOR (XO plus XDH) activity. Fluorometric assays were performed to calculate the production of isoxanthopterin. The activity was expressed as units/mg protein (tissue homogenate) using buttermilk XO (Merck Millipore, Billerica, MA) as standard. Quantified XO and XDH activities were expressed relative to the amount of total protein. Total protein concentration was measured using the Pierce BCA Protein Assay Kit (Thermo Scientific Inc., Billerica, MA).

### Lipid peroxidation and hydrogen peroxide measurement

Levels of MDA and H_2_O_2_ were determined in plasma, adipose, liver and intestine, using Lipid Peroxidation Assay Kit (Abcam) and Hydrogen Peroxide Assay Kit (BioVision, Palo Alto, CA) according to the instructions provided by the respective manufacturer.

### Intraperitoneal glucose and insulin tolerance tests

After two weeks of daily stress, mice received intraperitoneal glucose tolerance test (GTT) and insulin tolerance test (ITT) using standard methods^4^. Briefly, for GTT, mice were fasted overnight and then challenged with D-glucose at 2 g/kg body weight (Sigma-Aldrich, St. Louis, MO), followed by serial measurements of blood glucose up to 120 min using a blood glucose level monitor (Glutest Ace, Sanwa Kagaku Kenkyusho Co., Nagoya, Japan). For ITT, mice were fasted for 16 hours before testing. Insulin (0.75 U/kg, Actrapid Penfill, NovoNordisk, Copenhagen, Denmark) was injected intraperitoneally, and blood glucose was measured.

### Statistical analysis

Data are expressed as mean ± SD. Differences between groups were assessed by the Student’s t-test. Differences between quantitative data of different groups were analyzed by Fisher’s protected least significant differences (PLSD) test of one-way analysis of variances (ANOVA). The relationship between XOR activity and population of CD11b positive cells was analyzed by Pearson’s correlation coefficient. Differences between groups were considered significant when P was < 0.05.

## Electronic supplementary material

supplemental figure1

## References

[1] Katz JL, Weiner H (1972). Psychosomatic considerations in hyperuricemia and gout. Psychosomatic medicine.

[2] Uetani M (2006). A longitudinal study of the influence of shift work on serum uric acid levels in workers at a telecommunications company. Occupational medicine.

[3] Cheung BM, Li C (2012). Diabetes and hypertension: is there a common metabolic pathway?. Curr Atheroscler Rep.

[4] Uchida Y (2012). Stress augments insulin resistance and prothrombotic state: role of visceral adipose-derived monocyte chemoattractant protein-1. Diabetes.

[5] Yamamoto K (2002). Plasminogen activator inhibitor-1 is a major stress-regulated gene: implications for stress-induced thrombosis in aged individuals. Proceedings of the National Academy of Sciences of the United States of America.

[6] Tamura Y (2008). Inhibition of CCR2 ameliorates insulin resistance and hepatic steatosis in db/db mice. Arteriosclerosis, thrombosis, and vascular biology.

[7] Miller MW, Sadeh N (2014). Traumatic stress, oxidative stress and post-traumatic stress disorder: neurodegeneration and the accelerated-aging hypothesis. Molecular psychiatry.

[8] Hayashi M (2014). Angiotensin II receptor blocker ameliorates stress-induced adipose tissue inflammation and insulin resistance. PloS one.

[9] Yisireyili M (2016). Dipeptidyl peptidase- IV inhibitor alogliptin improves stress-induced insulin resistance and prothrombotic state in a murine model. Psychoneuroendocrinology.

[10] Boueiz A, Damarla M, Hassoun PM (2008). Xanthine oxidoreductase in respiratory and cardiovascular disorders. American journal of physiology. Lung cellular and molecular physiology.

[11] Lima WG, Martins-Santos ME, Chaves VE (2015). Uric acid as a modulator of glucose and lipid metabolism. Biochimie.

[12] Tsushima Y (2013). Uric acid secretion from adipose tissue and its increase in obesity. The Journal of biological chemistry.

[13] Page S (1998). Xanthine oxidoreductase in human mammary epithelial cells: activation in response to inflammatory cytokines. Biochimica et biophysica acta.

[14] Furukawa S (2004). Increased oxidative stress in obesity and its impact on metabolic syndrome. The Journal of clinical investigation.

[15] Battelli MG, Polito L, Bolognesi A (2014). Xanthine oxidoreductase in atherosclerosis pathogenesis: not only oxidative stress. Atherosclerosis.

[16] Pacher P, Nivorozhkin A, Szabo C (2006). Therapeutic effects of xanthine oxidase inhibitors: renaissance half a century after the discovery of allopurinol. Pharmacological reviews.

[17] Takeshita K (2004). Sinoatrial node dysfunction and early unexpected death of mice with a defect of klotho gene expression. Circulation.

[18] Beckman JS, Parks DA, Pearson JD, Marshall PA, Freeman BA (1989). A sensitive fluorometric assay for measuring xanthine dehydrogenase and oxidase in tissues. Free radical biology & medicine.

[19] Feoli AM, Macagnan FE, Piovesan CH, Bodanese LC, Siqueira IR (2014). Xanthine oxidase activity is associated with risk factors for cardiovascular disease and inflammatory and oxidative status markers in metabolic syndrome: effects of a single exercise session. Oxidative medicine and cellular longevity.

[20] Fleshner M (2013). Stress-evoked sterile inflammation, danger associated molecular patterns (DAMPs), microbial associated molecular patterns (MAMPs) and the inflammasome. Brain, behavior, and immunity.

[21] Reber SO (2007). Adrenal insufficiency and colonic inflammation after a novel chronic psycho-social stress paradigm in mice: implications and mechanisms. Endocrinology.

[22] Borghi, C. et al. Serum uric acid and the risk of cardiovascular and renal disease. *Journal of hypertension* **33**, 1729–1741, discussion 1741, doi:10.1097/HJH.0000000000000701 (2015). 10.1097/HJH.0000000000000701 26136207

[23] Maiuolo J, Oppedisano F, Gratteri S, Muscoli C, Mollace V (2016). Regulation of uric acid metabolism and excretion. International journal of cardiology.

[24] Kather H (1990). Beta-adrenergic stimulation of adenine nucleotide catabolism and purine release in human adipocytes. The Journal of clinical investigation.

[25] Baldwin W (2011). Hyperuricemia as a mediator of the proinflammatory endocrine imbalance in the adipose tissue in a murine model of the metabolic syndrome. Diabetes.

[26] Suganami T, Nishida J, Ogawa Y (2005). A paracrine loop between adipocytes and macrophages aggravates inflammatory changes: role of free fatty acids and tumor necrosis factor alpha. Arteriosclerosis, thrombosis, and vascular biology.

[27] Takeshita K, Murohara T (2014). Does angiotensin receptor blockade ameliorate the prothrombotic tendency in hypertensive patients with atrial fibrillation? Breaking the vicious cycle. Hypertension research: official journal of the Japanese Society of Hypertension.

[28] Creager MA, Luscher TF, Cosentino F, Beckman JA (2003). Diabetes and vascular disease: pathophysiology, clinical consequences, and medical therapy: Part I. Circulation.

[29] Cimmino G (2015). Reactive oxygen species induce a procoagulant state in endothelial cells by inhibiting tissue factor pathway inhibitor. Journal of thrombosis and thrombolysis.

[30] Han CY (2016). Roles of Reactive Oxygen Species on Insulin Resistance in Adipose Tissue. Diabetes & metabolism journal.

[31] Kauppinen A, Suuronen T, Ojala J, Kaarniranta K, Salminen A (2013). Antagonistic crosstalk between NF-kappaB and SIRT1 in the regulation of inflammation and metabolic disorders. Cellular signalling.

[32] Ruan H (2002). Profiling gene transcription *in vivo* reveals adipose tissue as an immediate target of tumor necrosis factor-alpha: implications for insulin resistance. Diabetes.

[33] Benito M (2011). Tissue-specificity of insulin action and resistance. Arch Physiol Biochem.

[34] Wang C (2007). Adiponectin sensitizes insulin signaling by reducing p70 S6 kinase-mediated serine phosphorylation of IRS-1. The Journal of biological chemistry.

[35] Berry CE, Hare JM (2004). Xanthine oxidoreductase and cardiovascular disease: molecular mechanisms and pathophysiological implications. The Journal of physiology.

[36] Berk M (2013). Aspirin: a review of its neurobiological properties and therapeutic potential for mental illness. BMC medicine.

[37] Aoyama T (2009). gamma-Secretase inhibitor reduces diet-induced atherosclerosis in apolipoprotein E-deficient mice. Biochemical and biophysical research communications.

[38] Nomura J (2014). Xanthine oxidase inhibition by febuxostat attenuates experimental atherosclerosis in mice. Scientific reports.

