# Supplementary material for: Xanthine oxidase inhibition by febuxostat attenuates stress-induced hyperuricemia, glucose dysmetabolism, and prothrombotic state in mice
Source: Sci Rep. 2017 Apr 28;7:1266. doi: 10.1038/s41598-017-01366-3 (PMC5430858; doi:10.1038/s41598-017-01366-3)
Supplement: Supplementary file 1 — supplemental figure1 [file 41598_2017_1366_MOESM1_ESM.pdf]

Online Supplemental Figure and Figure Legend

Xanthine oxidase inhibition by febuxostat attenuates stress-induced hyperuricemia, glucose dysmetabolism, and prothrombotic state in mice

Maimaiti Yisireyili[1], Motoharu Hayashi[1], Hongxian Wu[1,6], Yasuhiro Uchida[1], Koji Yamamoto[3], Ryosuke Kikuchi[2], Mohammad Shoaib Hamrah[1], Takayuki Nakayama[5], Xian Wu Cheng[1], Tadashi Matsushita[2 3], Shigeo Nakamura[4], Toshimitsu Niwa[7], Toyoaki Murohara[1], and Kyosuke Takeshita[1,2]

[1]Department of Cardiology, Nagoya University Graduate School of Medicine, Nagoya, Japan. Departments of [2]Clinical Laboratory, [3]Blood Transfusion, and [4]Pathology, Nagoya University Hospital, Nagoya, Japan. [5]Department of Blood Transfusion, Aichi Medical University Hospital, Nagakute, Japan. [6] Department of Cardiology, Shanghai General Hospital, Shanghai Jiao Tong University School of Medicine, Shanghai, China. [7]Faculty of Health and Nutrition, Shubun University, Ichinomiya, Aichi, Japan.

Running title: Febuxostat improves stress-induced metabolic abnormalities.

Correspondence to: A/Prof. Kyosuke Takeshita, MD, PhD, FAHA,

Department of Cardiology Nagoya University Graduate School of Medicine, 65 Tsurumai-cho Nagoya, Aichi 466-8550, Japan.

Tel: +81 52 744 2147; Fax: +81 52 744 2138

E-mail: kyousuke@med.nagoya-u.ac.jp

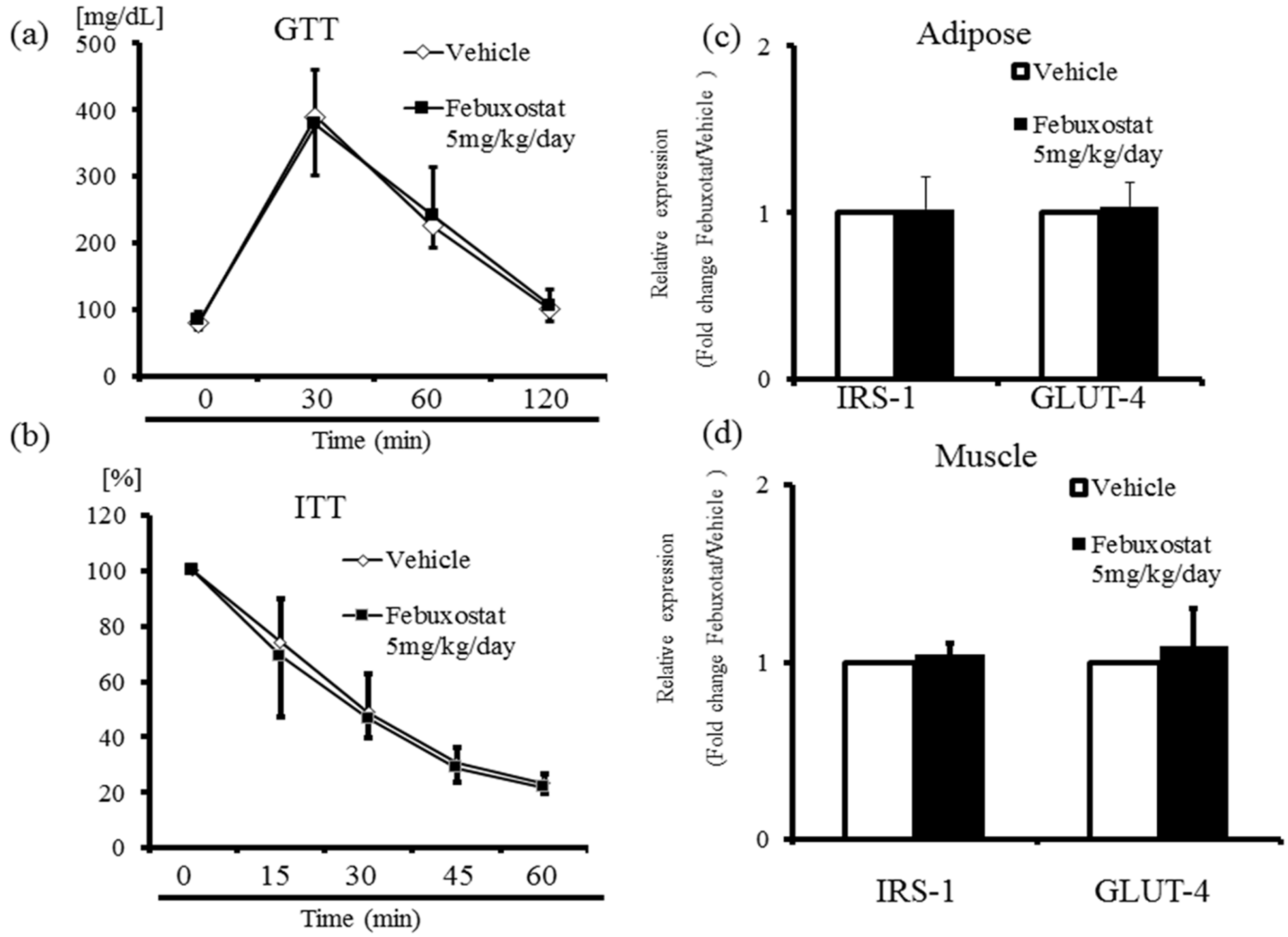
(a)
[mg/dL]
GTT
Vehicle
Febuxostat 5mg/kg/day
500
400
300
200
100
0
0
30
60
120
Time (min)
(b)
[%]
ITT
Vehicle
Febuxostat 5mg/kg/day
120
100
80
60
40
20
0
0
15
30
45
60
Time (min)
(c)
Adipose
Vehicle
Febuxostat 5mg/kg/day
Relative expression (Fold change Febuxotat/Vehicle)
2
1
0
IRS-1
GLUT-4
(d)
Muscle
Vehicle
Febuxostat 5mg/kg/day
Relative expression (Fold change Febuxotat/Vehicle)
2
1
0
IRS-1
GLUT-4

Figure legend

Supplemental Figure 1. Febuxostat did not alter glucose metabolism in unstressed mice.

Intraperitoneal glucose tolerance (GTT) and insulin tolerance tests (ITT) were performed in unstressed mice with the vehicle- or febuxostat- (5 mg/kg/day) treatment. The mRNA expression levels of IRS-1 and GLUT4 in inguinal adipose tissue and skeletal muscle (adductor muscle) were analyzed by quantitative RT-PCR. Data were analyzed by Student's t-test and displayed as mean±SD of 8 mice per group. **(a)** Glucose tolerance was comparable between the vehicle- and febuxostat-treated mice. **(b)** Insulin tolerance was comparable between the vehicle- and febuxostat-treated mice. Quantitative analysis of IRS-1 and GLUT4 expression levels in inguinal adipose tissue **(c)** and skeletal muscle (adductor muscle) **(d)** of unstressed mice treated with vehicle or febuxostat (5 mg/kg/day). All the data were comparable between the vehicle- and febuxostat-treated mice without stress.

Online Supplemental Figure Legend. Febuxostat improves stress-induced metabolic abnormalities state. 5
